# Deep Learning-Based High-Resolution Magnetic Resonance Angiography (MRA) Generation Model for 4D Time-Resolved Angiography with Interleaved Stochastic Trajectories (TWIST) MRA in Fast Stroke Imaging

**DOI:** 10.3390/diagnostics14111199

**Published:** 2024-06-06

**Authors:** Bo Kyu Kim, Sung-Hye You, Byungjun Kim, Jae Ho Shin

**Affiliations:** Department of Radiology, Anam Hospital, Korea University College of Medicine, #126-1, 5-Ka Anam-dong, Sungbuk ku, Seoul 136-705, Republic of Korea; stingray0379@naver.com (B.K.K.); cardillo@hanmail.net (B.K.); shinjh@kumc.or.kr (J.H.S.)

**Keywords:** MR angiography, deep neural network, denoising

## Abstract

Purpose: The purpose of this study is to improve the qualitative and quantitative image quality of the time-resolved angiography with interleaved stochastic trajectories technique (4D-TWIST-MRA) using deep neural network (DNN)-based MR image reconstruction software. Materials and Methods: A total of 520 consecutive patients underwent 4D-TWIST-MRA for ischemic stroke or intracranial vessel stenosis evaluation. Four-dimensional DNN-reconstructed MRA (4D-DNR) was generated using commercially available software (SwiftMR v.3.0.0.0, AIRS Medical, Seoul, Republic of Korea). Among those evaluated, 397 (76.3%) patients received concurrent time-of-flight MRA (TOF-MRA) to compare the signal-to-noise ratio (SNR), image quality, noise, sharpness, vascular conspicuity, and degree of venous contamination with a 5-point Likert scale. Two radiologists independently evaluated the detection rate of intracranial aneurysm in TOF-MRA, 4D-TWIST-MRA, and 4D-DNR in separate sessions. The other 123 (23.7%) patients received 4D-TWIST-MRA due to a suspicion of acute ischemic stroke. The confidence level and decision time for large vessel occlusion were evaluated in these patients. Results: In qualitative analysis, 4D-DNR demonstrated better overall image quality, sharpness, vascular conspicuity, and noise reduction compared to 4D-TWIST-MRA. Moreover, 4D-DNR exhibited a higher SNR than 4D-TWIST-MRA. The venous contamination and aneurysm detection rates were not significantly different between the two MRA images. When compared to TOF-MRA, 4D-CE-MRA underestimated the aneurysm size (2.66 ± 0.51 vs. 1.75 ± 0.62, *p* = 0.029); however, 4D-DNR showed no significant difference in size compared to TOF-MRA (2.66 ± 0.51 vs. 2.10 ± 0.41, *p* = 0.327). In the diagnosis of large vessel occlusion, 4D-DNR showed a better confidence level and shorter decision time than 4D-TWIST-MRA. Conclusion: DNN reconstruction may improve the qualitative and quantitative image quality of 4D-TWIST-MRA, and also enhance diagnostic performance for intracranial aneurysm and large vessel occlusion.

## 1. Introduction

Cranio-cervical artery evaluation through MR angiography is essential when deciding whether to perform endovascular thrombectomy in patient with large vessel occlusion (LVO) [1]. However, time-of-flight (TOF) MRA has the disadvantage of a longer acquisition time in patients with hyperacute stroke [2,3,4]. In contrast to TOF-MRA, contrast-enhanced MRA (CE-MRA) has the advantages of a shorter acquisition time and larger field of view (FOV) in patients suspected of acute ischemic stroke (AIS) [5]. Since the patient’s prognosis is closely related to the onset to recanalization time, it is important to shorten the acquisition time. Additionally, evaluating aortic arch anatomy through a larger FOV helps plan endovascular treatment. Recently, dynamic contrast MRA, also called 4-dimensional contrast-enhanced MRA (4D-CE-MRA) was applied in a patient with AIS to evaluate the presence of LVO and collateral status with a reasonable acquisition time [6].

With a stronger magnetic field (i.e., 3T), technical advancement in receive coil design, and optimized sequences, CE-MRA has shown reliable results in the diagnosis of LVO [7,8]. The time-resolved angiography with interleaved stochastic trajectories technique (4D-TWIST-MRA) has recently achieved higher temporal resolution than conventional 4D-CE-MRA [9,10]. However, the major drawback of 4D-TWIST-MRA is its relatively lower signal-to-noise ratio (SNR) compared to TOF-MRA. The spatial resolution of CE-MRA is lower than TOF-MRA due to its requirements of extended coverage and acquisition speed [11]. This phenomenon is even more noticeable in 4D-TWIST-MRA because multiple phases of images are acquired during a much shorter period of time. As the demand for distal medium vessel occlusion treatment beyond LVO increases, improving image quality plays an important role in patient selection [12]. Furthermore, incidental unruptured intracranial aneurysms are often diagnosed in patients evaluated by MRA, and an improvement in the image quality of 4D-CE-MRA is important for the accurate diagnosis of a patient’s vascular diseases [13].

Recently, various models of a deep neural network (DNN) have been developed for the enhancement of image resolution [14,15], reducing contrast agent usage [16], or reducing the image acquisition time with undersampling [17]. Several reports have provided encouraging results in improving MRA quality with DNN reconstruction [15,18]. Thus, we have investigated the clinical feasibility of DNN-generated 4D-CE-MRA using a commercially available DNN-based MR image reconstruction software (SwiftMR, v.3.0.0.0. AIRS Medical, Seoul, Republic of Korea).

## 2. Materials and Methods

### 2.1. Study Population

This retrospective study was approved by the institutional review board of our institution (IRB No. 2023AN0228), and the requirement for informed consent was waived. We reviewed consecutive patients who underwent MRA from April 2021 to January 2022. For patients clinically suspected of AIS, only 4D-TWIST-MRA was performed, while for patients not in the acute stroke setting, both 4D-TWIST-MRA and TOF-MRA were performed.

### 2.2. Image Acquisition

MR images were acquired using two 3.0 T MRI scanners (Skyra and Prisma, Siemens Healthineers, Erlangen, Germany) with a 64-channel head and neck coil. In patients without acute neurological symptoms, TOF-MRA and 4D-CE-MRA were obtained in a single session. The imaging parameters for TOF-MRA were: TR = 22–23 ms, TE = 3–4.02 ms; flip angle = 18°; number of excitation (NEX) = 1; bandwidth = 76.8–82.8 kHz; field of view (FOV) = 172 × 230 mm; matrix = 448–512 × 235–303; number of slabs = 11; section thickness = 0.5–0.6 mm; number of slices in one slab = 32; thickness of one slab = 16 mm; slab overlap = 5.5 mm; total acquisition time = 5 min 25–46 s; reconstructed voxel size = 0.6 × 0.6 × 0.6 mm; and number of maximum intensity projection (MIP) = 40. Four-dimensional CE-MRA was performed with a time-resolved MRA sequence (TWIST; Siemens Healthcare, Erlangen, Germany). The parameters used for 4D-TWIST-MRA were: TR = 2.87 ms; TE = 1.06 ms; flip angle 21°; NEX 1; bandwidth 102.4 kHz; FOV 300 × 400 mm; matrix 448 × 218; section thickness 0.85 mm; reconstructed voxel size = 0.9 × 0.9 × 0.8 mm; temporal resolution = 1.25 s; and total acquisition time = 57.6 s. For 4D-CE-MRA, automatic injection of 0.2 mL/kg gadoteridol (ProHance, Bracco, Milan, Italy) was followed by 30 mL saline. Both TOF-MRA and 4D-CE-MRA MIP were automatically generated using the same method with Siemens MR workstation’s 3D software. Four-dimensional CE-MRA MIP data were obtained for 40.6 s of the arterial phase image with the subtraction of non-enhancement data.

### 2.3. Denoising of 4D-CE-MRA with SwiftMR

A commercially available DNN-based MR image reconstruction software (SwiftMR, v.3.0.0.0. AIRS Medical, Seoul, Republic of Korea) was used in this study to obtain denoised MR images (4D-DNR). The software performs denoising and spatial resolution enhancements in the Digital Imaging and Communications in Medicine (DICOM) domain as a post-processing step. The algorithm was developed utilizing a 2D U-net structure [19]. The model is composed of cascading 18 convolutional blocks, four max-pooling layers, four up-sampling layers, four feature concatenations, and three convolutional layers, and data consistency is enforced in each layer. The network was trained by 31,865 MR image series and an internal validation was conducted using 3540 MR image series. The MR images used for training and validating the model were collected from multiple hospitals in South Korea. Both 2D and 3D acquisitions, along with multiple contrasts, imaging sequences, field strengths, coil configurations, and different anatomical localizations including the brain, head, and neck were included considering the clinical environment.

For algorithm training, MR images with a high SNR and high spatial resolution were utilized as label data paired with low SNR, low-resolution images as input. Various low-resolution k-space data for the undersampling technique were provided by each MRI vendor (for example, uniform, random, elliptical, partial Fourier undersampling, etc.). Image up-sampling was performed through deep learning based on context-enhanced U-Net. Therefore, the model is capable of spatial resolution enhancement as well as recognizing and reducing noise in the images. Furthermore, the structural similarity index (SSIM) between the input and label images was used for defining the model’s loss function. The model was optimized with Adam [20] over 20 epochs using a batch size of four at a learning rate of 10-3, decaying to 10-4, and the network was trained by four NVIDIA Tesla V100 GPUs with 32 GB memory (NVIDIA Corporation, Santa Clara, CA, USA). All images utilized for this reconstruction model training and validation were exclusive from those of this study. The detailed model development process and clinical application scenarios are provided in Jeong et al. [21].

### 2.4. Image Interpretation

#### Image Quality Comparison between TOF-MRA, 4D-TWIST-MRA, and 4D-DNR

**Figure 1:**
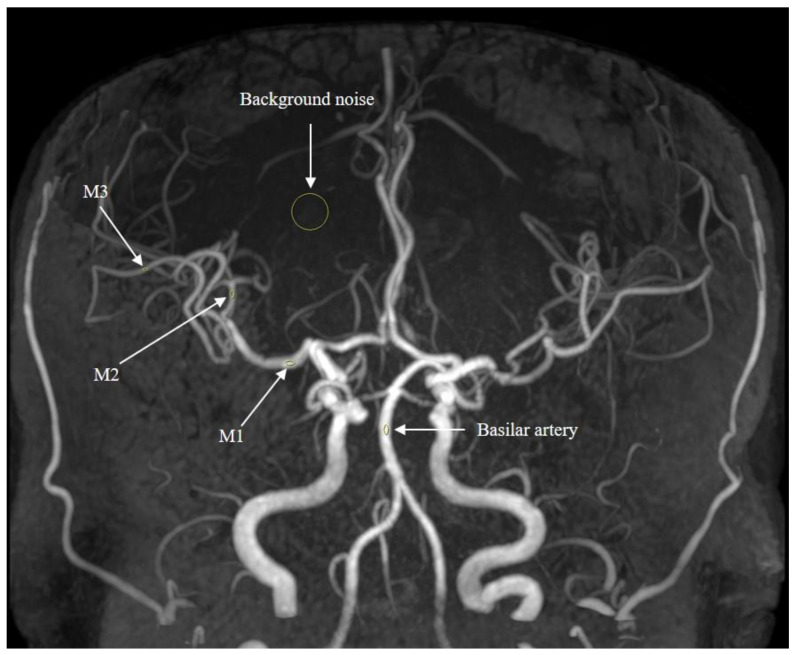
ROI measurement. Signal intensity values were measured by placing circular region of interest on M1, M2, M3, basilar artery, and background.

Qualitative and quantitative image quality analysis for each MRA were independently performed by two radiologists (SHY and BKK, 13 and 11 years of experience in neuroradiology). Any discrepancies were resolved by consensus. The mean values were utilized for quantitative analysis. Each image set was comprised of MIP images (AP and lateral rotational views). Qualitative analysis was conducted based on a 5-point Likert scale for overall image quality, noise, sharpness, and degree of venous contamination. The SNRs of M1, M2, M3, basilar artery, and background were calculated as the mean signal intensity (SI) of the lesion divided by the standard deviation of the background SI (noise). Circular regions of interest were delineated at the lumen of the vessel showing the highest SI on the MIP image (Figure 1).

### 2.5. Clinical Usability Assessment

#### Reading Confidence and Time for Decision of LVO for AIS Patients

To evaluate the diagnosis of LVO in patients with AIS, two neuroradiologists (13 years of experience and 11 years of experience of neuroradiology, and 8 years of experience in neurointervention for the latter) assessed LVO’s presence or absence. The confidence in diagnosis and time to diagnosis were recorded for both 4D-TWIST-MRA and 4D-DNR. The confidence level of diagnosis was evaluated based on a 5-point Likert scale as follows, 1: not confident at all, 2: slightly confident, 3: somewhat confident, 4: fairly confident, 5: completely confident.

### 2.6. Aneurysm Assessment

For patients diagnosed with unruptured intracranial aneurysms based on TOF-MRA findings, the detection rate of the aneurysm was evaluated on both 4D-TWIST-MRA and 4D-DNR. Two radiologists, blinded to clinical information, assessed the presence of aneurysms in the MIP image set. Additionally, when an aneurysm was found in the MIP set of 4D-TWIST-MRA and 4D-DNR, the maximum diameter of the aneurysm was measured and compared with TOF-MRA.

### 2.7. Statistical Analysis

All data were presented as a mean with standard deviation, or a number with percentages. Continuous variables with normal distribution were analyzed with Student t-tests. Continuous variables without normal distribution were analyzed with the Mann–Whitney U test. Categorical variables were analyzed with the Chi square test or Fisher exact test. Analysis of variance (ANOVA) was used to compare image qualities and SNR for three MRA modalities, and post hoc analyses using Bonferroni correction were performed to assess the differences among the three groups. All *p*-values are 2-sided. Statistical analyses were carried out using SPSS statistics version 22.0 (IBM Corporation, Armonk, NY, USA).

## 3. Results

**Figure 2:**
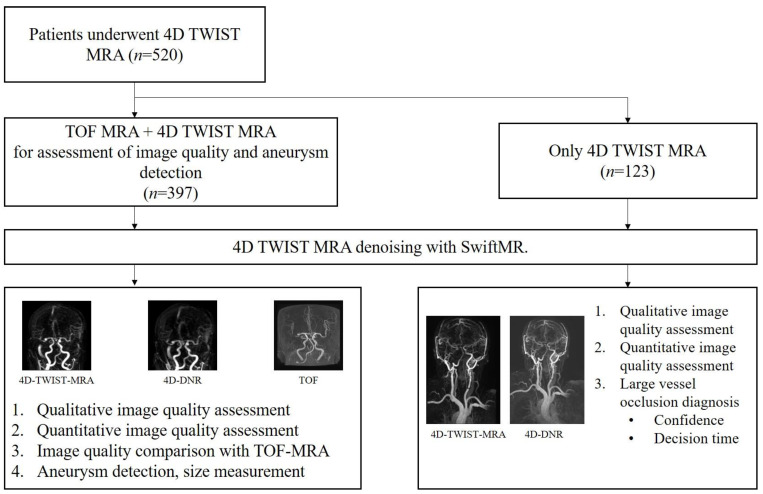
Schematic flow of study design. Four-dimensional DNR images were obtained using SwiftMR software based on the 4D-TWIST-MRA. One hundred and twenty-three patients underwent 4D-TWIST-MRA under suspicion of large vessel occlusion. The remaining 397 patients underwent concurrent TOF-MRA for intracranial artery evaluation. The clinical usefulness of each MRA modality was evaluated according to clinical setting.

A total of 520 patients underwent 4D-TWIST-MRA. Among them, 123 patients (23.7%) were suspected of AIS. For these patients, concurrent TOF-MRA was not performed, and qualitative and quantitative image quality were evaluated for both 4D-TWIST-MRA and 4D-DNR. The remaining 397 (76.3%) patients underwent MRA in a non-acute clinical setting. A schematic flow of the study design is presented in Figure 2.

### 3.1. Image Quality Assessment

**Figure 3:**
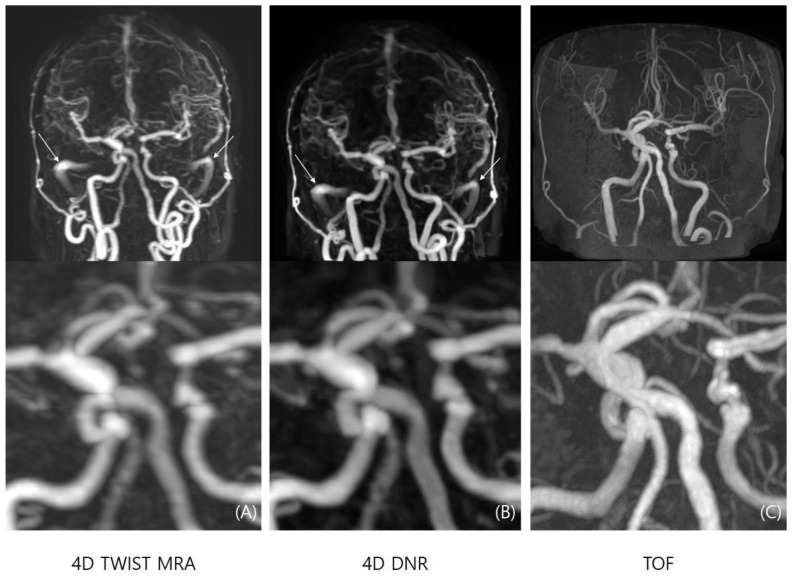
Maximum intensity projection images of 4D-TWIST-MRA (**A**), 4D-DNR (**B**), and TOF-MRA (**C**). Sharpness and vascular conspicuity of 4D-DNR were improved, and background noise was significantly reduced after denoising. Venous contamination (e.g., high signal intensity in bilateral sigmoid sinus, arrows in (**A**,**B**)) was not changed after denoising.

The results for the quantitative and qualitative image quality among the three MRA MIP series are summarized in Table 1. The SNR is usually higher in the M1 segment and basilar artery, with the SNR of each segment decreasing toward the distal branches in all sequences. The SNR is highest in TOF-MRA, followed by 4D-DNR and 4D-TWIST-MRA in all ROIs (SNR for M1, TOF-MRA: 62.4 ± 33.0; 4D-TWIST-MRA: 21.4 ± 7.0; 4D-DNR: 30.5 ± 12.4, *p* = 0.001). Background noise is significantly higher in 4D-TWIST-MRA, and is significantly reduced after denoising (19.9 ± 14.6 vs. 9.37 ± 6.7, *p* = 0.001). However, the degree of vascular contamination is not significantly changed (3.33 vs. 3.18, *p*-value 0.679). When compared with TOF-MRA and 4D-DNR, there is no significant difference in background noise (11.3 ± 5.6 vs. 9.4 ± 6.7, *p* = 0.813) (Figure 3).

**Figure 4:**
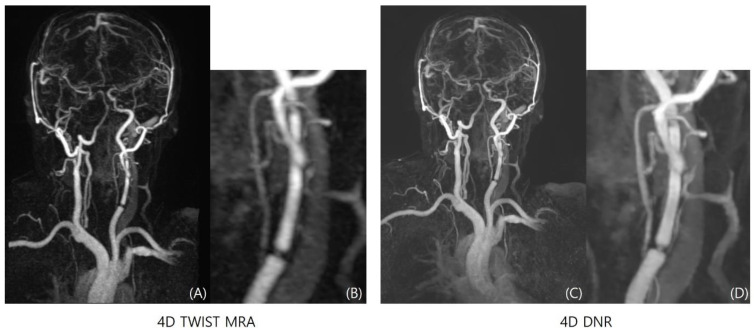
Comparison between 4D-TWIST-MRA (**A**,**B**) and 4D-DNR (**C**,**D**). Stent placement state at left carotid artery. In subjective evaluation, 4D-DNR showed better overall image quality, vascular conspicuity, and sharpness.

Qualitative indicators including overall image quality (3.25 ± 0.42 vs. 2.55 ± 0.52, *p* = 0.040), noise (3.40 ± 0.52 vs. 2.68 ± 0.62, *p* = 0.016), vascular conspicuity (3.27 ± 0.52 vs. 2.80 ± 0.50, *p* = 0.037), and sharpness (3.18 ± 0.51 vs. 2.12 ± 0.33, *p* = 0.035) are significantly improved in the 4D-DNR sequence than 4D-TWIST-MRA (Figure 4).

### 3.2. Aneurysm Detection

**Figure 5:**
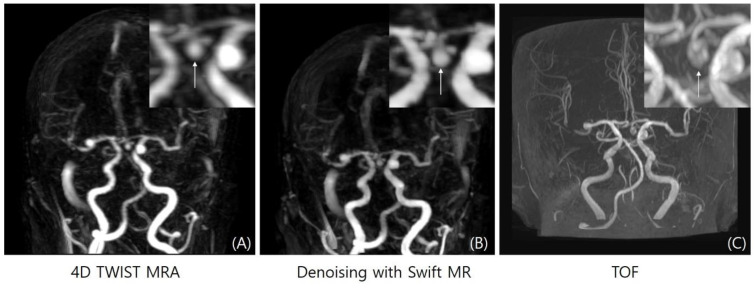
Comparison of aneurysm size measurement. The sizes of anterior communicating artery aneurysm (arrows) were measured as 4.26 mm, 4.67 mm, and 5.23 mm in 4D-TWIST MRA (**A**), 4D-DNR (**B**), and TOF-MRA (**C**), respectively.

Based on TOF-MRA, a total of 54 aneurysms were detected in 38 patients (Table 2). Four-dimensional TWIST-MRA and 4D-DNR found 42 (77.8%) and 44 (81.5%) aneurysms, respectively. The detection rate was not significantly different in both modalities. The maximal diameters of aneurysms measured on 4D-TWIST-MRA and 4D-DNR were 2.10 ± 0.41 mm and 1.75 ± 0.62 mm, respectively. In 4D-TWIST-MRA, the size of the aneurysm was underestimated and showed a significant difference from TOF (1.75 ± 0.62 mm vs. 2.66 ± 0.51 mm, *p*-value 0.29). In comparison, the size of the aneurysm in 4D-DNR tended to be smaller than that in TOF, but there was no statistically significant difference (2.10 mm vs. 2.66 mm, *p*-value 0.327) (Figure 5).

### 3.3. LVO Evaluation

**Figure 6:**
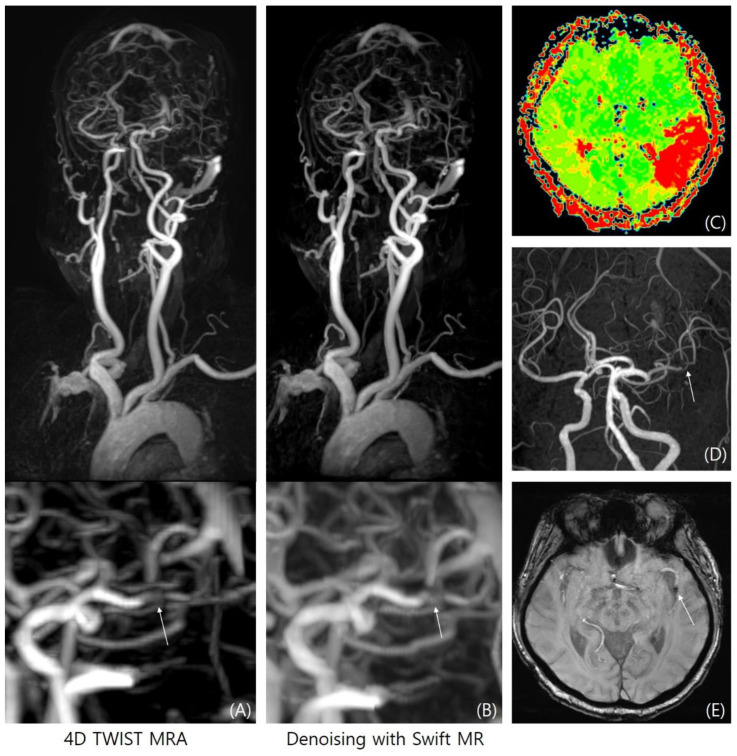
Large vessel occlusion detection. Four-dimensional TWIST-MRA (**A**) and 4D-DNR (**B**) showed filling defect at the left distal middle cerebral artery (arrows). Subsequent perfusion MRI (**C**), TOF-MRA (**D**), and susceptibility weighted image (**E**) confirmed thrombus (blooming artifact, arrow) with territorial perfusion delay.

In the acute clinical setting, where patients were suspected of AIS, a total of 25 (20.3%) patients showed intracranial LVO (Figure 6). The confidence level of LVO diagnosis was significantly higher in 4D-DNR than 4D-TWIST-MRA and the decision time tended to be shorter in 4D-DNR (Reader 1: 33.76 ± 11.0 s vs. 30.42 ± 9.6 s, *p* = 0.056; Reader 2: 31.61 ± 13.4 s vs. 27.15 ± 12.3 s, *p* = 0.042). The diagnosis performances of the two modalities are summarized in Table 3.

## 4. Discussion

The major findings of this study indicate that DNN-based denoising significantly improved the overall image quality, noise, sharpness, and vascular conspicuity of 4D-TWIST-MRA. Additionally, the improvement in the SNR may be beneficial in the diagnostic performance of aneurysm measurement, as well as in the diagnosis of LVO. Notably, the venous contamination and aneurysm detection rates showed no significant difference between 4D-TWIST-MRA and 4D-DNR.

Improving MRI image resolution using deep learning has emerged as a significant area of research in medical imaging. The 4D-TWIST-MRA technique has been accepted as a less invasive method for cranio-cervical artery evaluation compared to DSA, offering advantages such as providing time-resolved vascular information over single-phase MRA [22,23]. However, 4D-TWST-MRA often encounters limitations in resolution due to ghosting and blurring artifacts, which can affect the accuracy of diagnosis of smaller vessels [24]. Recent studies have demonstrated that 4D-TWIST-MRA with iterative reconstruction can improve the image quality for diagnosing arteriovenous malformation [25,26]. Nonetheless, recent advancements in deep learning have presented promising solutions for enhancing MRI image resolution, thereby improving the quality of medical imaging data. Deep learning-based reconstruction has shown impressive results in reducing image noise in both cross-sectional images and MIP images [18,27]. In our study, we were able to validate the effectiveness of DNN-based denoising in various clinical settings.

Another important consideration is that DNN-based reconstruction has demonstrated an improvement in diagnostic performance through the improvement of image quality, although its effectiveness can be influenced by the initial image quality. In CE-MRA, the contrast media of the venous structure remained delineated even after DNN reconstruction. Moreover, for small aneurysms that were not visible in the original 4D-TWIST MRA image, diagnosis remained challenging even after reconstruction, leading to no significant improvement in the aneurysm detection rate. However, it is worth noting that the undiagnosed aneurysms were mainly smaller than 2 mm. Despite this limitation, the size of the aneurysm was measured as being similar in 4D-DNR to that in TOF-MRA, which is expected to assist in establishing a treatment plan in clinical practice except for very small aneurysms.

Our previous study introduced the synthetic TOF-MRA generation from 4D-TWIST-MRA using a cycle-consistent generative adversarial network (CycleGAN; https://github.com/junyanz/CycleGAN, accessed on 20 May 2024) [28]. There are several major differences between the CycleGAN and the SwiftMR model. Since the previous model performed algorithm training between MIP images of 4D-TWIST-MRA and TOF-MRA, only images from the single arterial phase of 4D-TWIST-MRA can be applied in the CycleGAN model. SwiftMR-based 4D-DNR is a k-space-based model, which can be applied to all phases of 4D-TWIST-MRA. Further, TOF-MRA was usually performed for the evaluation of intracranial arteries, and image quality improvement for the neck vascular structure was not achieved in CycleGAN. In this study, we confirmed the image quality improvement with a wider FOV. Finally, there is a possibility of image distortion in the GAN-based mode because some images may be transformed into TOF-like images.

Our study has several limitations, including its retrospective and single-center design. External validation is essential to enhance the reliability of our findings. Additionally, the use of TOF-MRA as a comparison modality for 4D-TWIST-MRA and 4D-DNR may have introduced bias due to differences in MRA principles. A turbulent flow-related artifact or in-plane saturation artifact generated in TOF-MRA could have influenced our results. Nevertheless, it is noteworthy that the diagnostic performance of 4D-TWIST-MRA approached that of TOF-MRA due to 4D-DNR. Finally, our study evaluated clinical usefulness through MIP images, but further evaluation through cross-sectional images is necessary.

In conclusion, DNN-based image reconstruction represents a novel approach to improving the image quality of 4D-TWIST-MRA and diagnostic performance in clinical settings.

## Figures and Tables

**Table 1:** Comparison of overall image quality between TOF, 4D-TWIST-MRA, and 4D-DNR.

	TOF-MRA (*n* = 397)	4D-TWIST-MRA (*n* = 520)	4D-DNR (*n* = 520)	*p*-Value	TOF vs. 4D-TWIST-MRA	TOF vs. 4D-DNR	4D-TWIST-MRA vs. 4D-DNR
No.	395	395	395	N/A	N/A	N/A	N/A
Overall image quality	4.45 ± 0.52	2.55 ± 0.52	3.25 ± 0.42	0.001	0.001	0.010	0.040
Noise	4.46 ± 0.52	2.68 ± 0.62	3.40 ± 0.52	0.001	0.001	0.001	0.016
Sharpness	4.87 ± 0.34	2.12 ± 0.33	3.18 ± 0.51	0.001	0.001	0.001	0.035
Vascular conspicuity	3.52 ± 0.58	2.80 ± 0.50	3.27 ± 0.52	0.001	0.045	0.356	0.037
Venous contamination	5.00 ± 0.00	3.33 ± 0.80	3.18 ± 0.55	0.001	0.001	0.001	0.679
SI (M1)	558.9 ± 104.0	552.3 ± 350.2	561.7 ± 370.3	0.523	1.000	1.000	1.000
SI (M2)	420.3 ± 88.2	511.1 ± 373.6	575.3 ± 415.1	0.023	0.105	0.001	0.235
SI (M3)	325.4 ± 70.3	352.4 ± 231.2	434.6 ± 285.1	0.001	0.877	0.001	0.001
SI (BA)	659.4 ± 103.8	431.6 ± 271.0	570.9 ± 265.6	0.001	0.001	0.042	0.001
Background Noise	11.3 ± 5.6	19.9 ± 14.6	9.37 ± 6.7	0.001	0.001	0.813	0.001
SNR (M1)	62.4 ± 33.0	21.4 ± 7.0	30.5 ± 12.4	0.001	0.001	0.001	0.001
SNR (M2)	46.2 ± 23.3	14.1 ± 12.8	28.3 ± 12.9	0.001	0.001	0.001	0.001
SNR (M3)	34.0 ± 18.8	13.3 ± 11.0	18.0 ± 9.2	0.001	0.001	0.001	0.171
SNR (M4)	73.1 ± 35.0	20.5 ± 8.7	31.4 ± 9.5	0.001	0.001	0.001	0.021

N/A: not applicable.

**Table 2:** Comparison of aneurysm detection rate and size measurement.

	TOF-MRA	4D-TWIST-MRA	4D-DNR	*p*-Value	TOF vs. 4D-TWIST-MRA	TOF vs. 4D-DNR	4D-TWIST-MRA vs. 4D-DNR
Aneurysm detection	54 (100%)	42 (77.8%)	44 (81.5%)	0.001	<0.001	0.001	0.814
Aneurysm size	2.66 ± 0.51	1.75 ± 0.62	2.10 ± 0.41	0.033	0.029	0.327	0.251

**Table 3:** Comparison of acute ischemic stroke diagnosis.

	Reader 1			Reader 2		
	4D-TWIST-MRA	4D-DNR	*p*-Value	4D-TWIST-MRA	4D-DNR	*p*-Value
No.	123	123		123	123	
Confidence level of LVO diagnosis	3.92 ± 0.70	4.41 ± 0.58	0.007	3.82 ± 0.56	4.51 ± 0.61	0.003
Decision time (s)	33.76 ± 11.0	30.42 ± 9.6	0.056	31.61 ± 13.4	27.15 ± 12.3	0.042

## Data Availability

Data is contained within the article.
